# Genotypic Heterogeneity of *Orientia tsutsugamushi* in Scrub Typhus Patients and Thrombocytopenia Syndrome Co-infection, Myanmar

**DOI:** 10.3201/eid2608.200135

**Published:** 2020-08

**Authors:** Aye Marlar Win, Yen Thi Hai Nguyen, Yuri Kim, Na-Young Ha, Jun-Gu Kang, Hongil Kim, Bo San, Okkar Kyaw, Wah Win Htike, Dong-Ok Choi, Keun-Hwa Lee, Nam-Hyuk Cho

**Affiliations:** University of Medicine 1, Yangon, Myanmar (A.M. Win, O. Kyaw, W.W. Htike); Seoul National; University College of Medicine, Seoul, South Korea (Y.T.H. Nguyen, Y. Kim, N.-Y. Ha, J.-G. Kang, H. Kim, N.-H. Cho);; Private practitioner, Sagaing, Myanmar (B. San);; Bore Da Biotech, Seongnam, South Korea (D.-O. Choi);; Hanyang University College of Medicine, Seoul (K.-H. Lee);; Seoul National University Medical Research Center and Bundang Hospital, Seoul (N.-H. Cho)

**Keywords:** Bacteria, co-infection, differential diagnosis genotypes, mite-borne infections, Myanmar, Orientia tsutsugamushi, phylogenic analysis, scrub typhus, thrombocytopenia syndrome virus, tsutsugamushi, vector-borne infections, viruses

## Abstract

Serologic and molecular surveillance of serum collected from 152 suspected scrub typhus patients in Myanmar revealed *Orientia tsutsugamushi* of genotypic heterogeneity. In addition, potential co-infection with severe fever with thrombocytopenia syndrome virus was observed in 5 (3.3%) patients. Both scrub typhus and severe fever with thrombocytopenia syndrome are endemic in Myanmar.

Scrub typhus is a miteborne febrile illness caused by the bacterium *Orientia tsutsugamushi*, which is endemic in the Asia-Pacific region and a major cause of undifferentiated febrile disease (*1*). *O. tsutsugamushi* infections were documented in Myanmar during the 1940s (*2*). Since then, however, no report has described the prevalence and genetics of scrub typhus in Myanmar, although 2 studies, including 1 from 2017, identified scrub typhus as one of the primary infections causing acute febrile illness on the Thailand–Myanmar border (*3*,*4*). These results underscore the need for research on this vectorborne infection in Myanmar, including studies defining the genotypic diversity of *O. tsutsugamushi*. Lack of this information has been a serious obstacle to developing effective diagnostic methods and a vaccine for scrub typhus (*1*). 

The tickborne virus severe fever with thrombocytopenia syndrome virus (SFTSV), of the genus *Banyangvirus*, can cause hemorrhagic fever with a mortality rate of up to 40% (*5*). SFTSV infections are endemic in eastern Asia, and retrospective studies have confirmed its presence in China in 1996 (*6*), South Korea in 2000 (*7*), Japan in 2005 (*8*), and Vietnam in 2017 (*9*). In addition, mixed infection with SFTSV and *O. tsutsugamushi* has been detected in patients in South Korea, where both pathogens are endemic (*7*,*10*). These results further emphasize the urgent need for epidemiologic studies of vectorborne diseases in areas of endemicity to improve our ability to accurately differentiate febrile infectious diseases with atypical signs and symptoms during the initial stages so they can be promptly treated. Here, we used blood samples from suspected scrub typhus patients in Myanmar to investigate the serologic prevalence and genotypic diversity of *O. tsutsugamushi*. We also examined these patients for possible co-infection with SFTSV, which has been an emerging threat to public health in eastern Asia. 

## The Study

**Figure 1:**
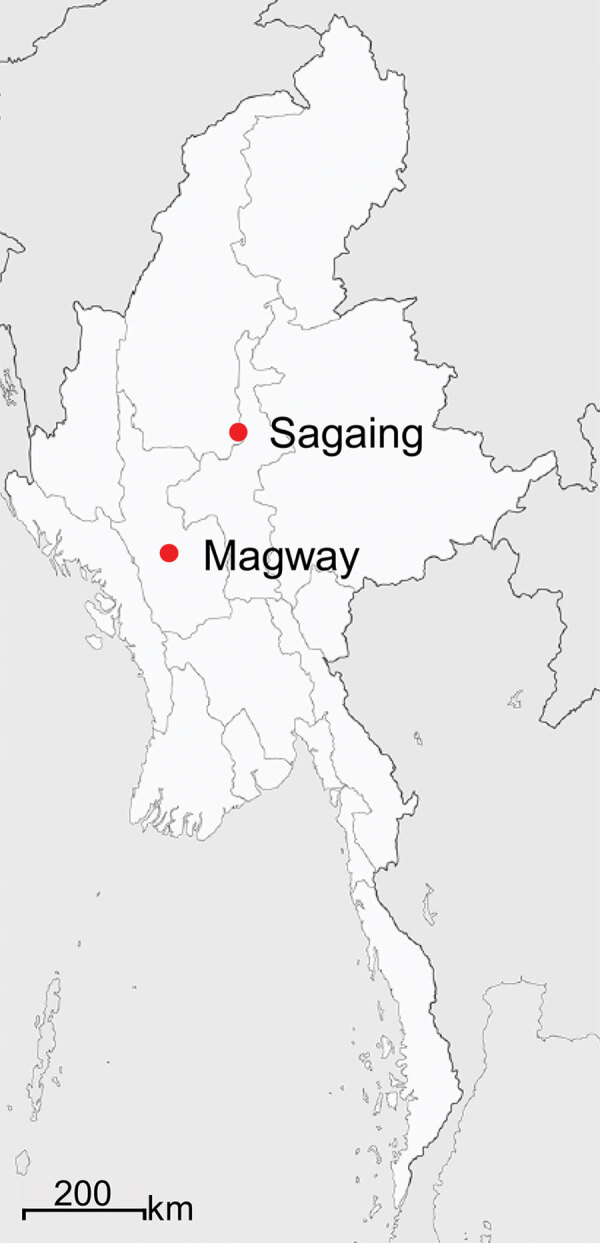
Locations in Sagaing and Magway Provinces in Myanmar, where suspected scrub typhus patients’ serum samples were collected for study of genotypic heterogeneity of *Orientia tsutsugamushi*.

To investigate the genotypic diversity of *O. tsutsugamushi* and potential co-infection with SFTSV in Myanmar, we collected whole blood samples from 152 clinically suspected scrub typhus patients (Table; Appendix Table 1) in Sagaing and Magway Provinces (Figure 1) during February 2018–January 2019. Mean age of the suspected scrub typhus patients was 27 ± 19.8 years (range of 2–73 years). We observed eschar, a selection criteria for scrub typhus, in 144 (94.7%) of the 152 patients. Mean fever duration was 6 days (SD ± 2.9 days). 

**Table:** Baseline characteristics and summary of serologic and molecular diagnosis of suspected scrub typhus patients enrolled in study of genotypic heterogeneity of *Orientia tsutsugamushi*, Myanmar

Category	Value
Age	
Age, y mean ± SD	27.0 ± 19.8
Age distribution, y	
≤10	38 (25.0)
11–20	38 (25.0)
21–30	23 (15.1)
31–40	10 (6.6)
41–50	17 (11.0)
51–60	16 (10.5)
≥61	10 (6.6)
Sex ratio, M:F (% male)	93/59 (61.2)
Clinical variables	
Fever duration, d, mean ±SD	6.1 ± 2.9
Eschar	144 (94.7)
Rash	3 (2.0)
Myalgia	25 (20)
Method of diagnosis of scrub typhus	
ICT	41/128 (32.0)
TSA56 IgG	36/128 (28.1)
ScaA IgG	25/128 (19.5)
IFA	138/152 (90.8)
*O. tsutsugamushi* IgG	119/152 (78.3)
*O. tsutsugamushi* IgM	90/152 (59.2)
PCR (*tsa56*)	9/152 (5.9)
Method of diagnosis of SFTSV	
RT-PCR	5/152 (3.3)

*Values are no. (%) patients or no. patients/no. tested (%) except as indicated. ICT, immunochromatography test; IFA, indirect immunofluorescence assay; RT-PCR, reverse transcription PCR; SFTSV, severe fever with thrombosis syndrome virus.

For initial serologic diagnosis of 128 serum samples, we used immunochromatography test strips coated with TSA56 and ScaA antigens, which revealed respective positive rates of 28.1% (36/128) and 19.5% (25/128); the overall positive rate was 32.0% (41/128). Of the 128 samples, 20 (15.6%) reacted with both antigens and 5 of 36 (13.9%) were positive for ScaA antigen only (Table; Appendix Figure 1), suggesting a potential applicability of ScaA antigen, when used simultaneously with TSA56 antigen, for the diagnosis of scrub typhus during the acute phase (*11*). To confirm serologic positivity against the bacterial antigen, we also conducted an indirect immunofluorescence assay using cells infected with *O. tsutsugamushi*, the standard method for diagnosing scrub typhus (*12*). Among the 152 serum samples we tested, results were positive (>1:40) for 119 (78.3%) for specific IgG and for 90 (59.2%) for IgM (Table; Appendix Table 1). Median titers of the positive serum samples were 1:640 for both IgG and IgM. Among the suspected scrub typhus patients, test results for 13 (8.6%) serum samples were negative for both IgG and IgM against *O. tsutsugamushi*. 

**Figure 2:**
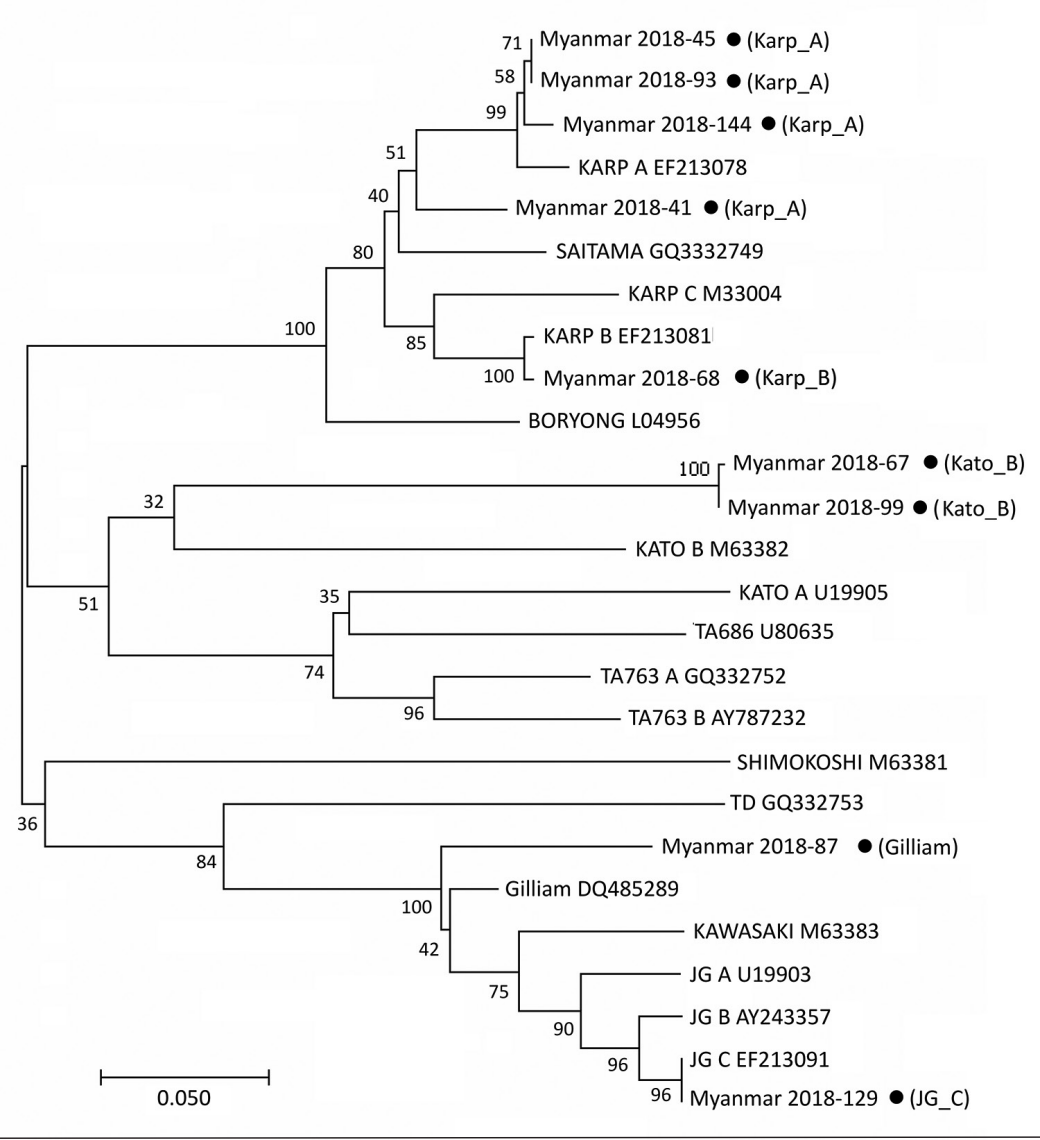
Phylogenetic tree constructed on the basis of *Orientia tsutsugamushi tsa56* gene sequences for scrub typhus patients in Myanmar (black dots) and reference sequences. The tree was constructed using the maximum likelihood method with MEGA7 (http://www.megasoftware.net). The *tsa56* gene sequences identified in this study are indicated by black circles and compared with 17 representative genotype sequences reported by a previous study (*1*). The percentage of replicate trees in which the associated genotypes clustered together in the bootstrap test (1,000 replicates) is shown next to the branches.

For molecular diagnosis of scrub typhus, we examined all the serum samples by PCR to confirm infection and identify the genotypes of *O. tsutsugamushi* in the patients in Myanmar. From the 152 serum samples, we detected specific PCR products in 9 (5.9%) and sequenced them for genotyping. We compared results of phylogenetic analysis of the 9 *tsa56* gene sequences with sequences from 17 protogenotypes (*1*), which revealed >5 genotypes, including Karp_A (4/9, 44.4%), Karp_B (1/9, 11.1%), Kato_B (2/9, 22.2%), Gilliam (1/9, 11.1%), and JG_C (1/9, 11.1%) (Figure 2). 

Finally, we used reverse transcription PCR analysis to investigate possible SFTSV infection in the patients (*7*,*9*,*13*). Among 152 patients’ serum samples, 5 (3.3%) were positive for the partial small (S) segment of the SFTSV RNA genome, indicating SFTSV infection. Results from phylogenetic analysis of the partial S segment sequences showed that 4 isolates were the same as those previously reported from Vietnam (*9*); 1 isolate differed by 1 base from the other 4 isolates (Appendix Figure 2), suggesting genetic homogeneity of SFTSV in southern Asia. Of note, 4 out of the 5 SFTSV-positive patients had eschar, and 4 were ˂15 years of age (Appendix Figure 2). Furthermore, 3 of them carried high titers (>1: 2560) of IgG, IgM, or both specific to *O. tsutsugamushi*, as measured by indirect immunofluorescence assay (Appendix Table 1), suggesting co-infection with scrub typhus. All patients were successfully treated and recovered, including the SFTSV-positive febrile patients, after 5–7 days of fever. 

## Conclusions

We observed a high prevalence of antibodies against *O. tsutsugamushi* in suspected scrub typhus patients in Myanmar, suggesting that scrub typhus, previously reported in the 1940s, remains prevalent in this country (*2*). Of note, a high prevalence of scrub typhus in children was confirmed (*4*); therefore, young children with febrile illness should be carefully observed for early diagnosis and treatment of scrub typhus. Because we were only able to examine serum samples collected from patients during the acute phase of infection and could not assess the rise of antibody titers in paired samples collected in convalescent phases, we were not able to confirm the exact rate of prevalence of scrub typhus in the suspected patients. The baseline levels of antibody titers against *O. tsutsugamushi* in healthy persons need to be assessed to determine the cutoff titer levels for diagnosing acute scrub typhus in the endemic region (*14*). 

In addition, genotyping *O. tsutsugamushi* revealed that >5 different genotypes are currently present and showed genetic heterogeneity in Myanmar. Moreover, we detected possible co-infection with SFTSV and *O. tsutsugamushi* in 5 patients. None of these patients had a history of travel abroad, and all live in the same village in Sagaing Province, suggesting that there may be hot spots for SFTSV infection. Co-infection with *O. tsutsugamushi* and SFTSV might be mediated by either simultaneous transmission from 2 different vectors each carrying 1 pathogen or by a single tick or mite species carrying both pathogens (*10*). Four of 5 SFTSV-positive patients were ˂15 years of age, and all 5 recovered within a week. Given that the disease severity of SFTS is associated with host age and the viral genotype (*15*), milder clinical symptoms observed in these patients might have been because of exposure at a younger age or prevalence of less virulent genotypes of SFTSV in Myanmar. Therefore, continuous surveillance of SFTS patients needs to be conducted, reporting detailed clinical manifestations and associated viral genotypes prevalent in the local area. In addition, more reliable differential diagnosis techniques and prevention and control measures are required for better clinical practices and outcomes in the endemic regions of multiple tickborne and miteborne pathogens. 

Appendix Additional information for genotypic heterogeneity of *Orientia tsutsugamushi* in scrub typhus patients and thrombocytopenia syndrome co-infection, Myanmar.
