# Supplementary material for: Genotypic Heterogeneity of Orientia tsutsugamushi in Scrub Typhus Patients and Thrombocytopenia Syndrome Co-infection, Myanmar
Source: Emerg Infect Dis. 2020 Aug;26(8):1878–81. doi: 10.3201/eid2608.200135 (PMC7392420; doi:10.3201/eid2608.200135)
Supplement: Appendix — Additional information for genotypic heterogeneity of Orientia tsutsugamushi in scrub typhus patients and thrombocytopenia syndrome co-infection, Myanmar. [file 20-0135-Techapp-s1.pdf]

Article DOI: https://doi.org/10.3201/eid2608.200135

Genotypic Heterogeneity of *Orientia tsutsugamushi* in Scrub Typhus Patients and Thrombocytopenia Syndrome Coinfection, Myanmar

Appendix

Study design and ethics

To investigate the genotypic diversity of *Orientia tsutsugamushi* and potential coinfection with SFTSV in Myanmar, whole blood samples were collected from clinically suspected scrub typhus patients in Sagaing and Magway provinces during February 2018–January 2019. We selected suspected scrub typhus patients if they presented ≥3 clinical manifestations, from among fever, headache, eschar, rash, myalgia, joint pain, lymphadenopathy, and respiratory symptoms (*1*). Specimens collected from 152 patients were transported to the University of Medicine 1 (Yangon, Myanmar) to prepare serum samples, which were stored at −80°C until used for testing at Seoul National University. This study was approved by the ethics review committee of the Department of Medical Research, Ministry of Health and Sports in Myanmar (Ethics/DMR/2018/134) and the institutional review boards of Seoul National University Hospital (IRB 1910–057–1069).

Serologic test

Immunochromatography test strips containing TSA56 and ScaA antigens from *O. tsutsugamushi* of Boryong, Gilliam, and Karp genotypes were manufactured (Bore Da Biotech Co., http://boreda.com/) and used, according to the manufacturer's instructions, to test serum samples for rapid diagnosis of scrub typhus. Results were visualized in 15 min after loading mixtures of a chasing buffer (200 µL) containing gold particles and 50 µL of patient serum on a sample pad. For immunofluorescence assay, we serially diluted serum samples from 1:40 to 1:10240 in phosphate buffered saline and incubated with pooled L929 cells infected with three genotypes of *O. tsutsugamushi* (Karp, Gilliam and Boryong), as previously described (*2*).

Amplification of nucleic acids and sequence analysis

From the serum samples, we extracted total DNA, using DNeasy Blood and Tissue kit, or viral RNA, using QIAamp viral RNA Mini kit (both from QIAGEN, https://www.qiagen.com/br/), according to the manufacturer's instructions. For molecular diagnosis, we performed PCR to amplify the *tsa56* gene of *O. tsutsugamushi* using 2 sets of primers (forward primer 1: GATCAAGCTTCCTCAGCCTACTATAATGCC, reverse primer 1: CGACAGATGC ACTATTAGGC, forward primer 2: TAGTGCAATGTCTGCGTTGTCGTTGCC, reverse primer 2: ACGCTGCAATTTAACAAGATCTTTATATAACT). We sequenced the amplified DNA fragments using the same primer sets and the partial sequences of the *O. tsutsugamushi tsa56* genes were deposited in GenBank under accession nos. MN913341 to MN913349.

To detect SFTSV RNA, we performed reverse transcription PCR to amplify the partial small (S) segment of the viral RNA from the serum samples and confirm SFTSV infection (*3*). We sequenced the PCR products using the BigDye Terminator Cycle Sequencing Kit (Perkin Elmer Applied Biosystems, https://www.perkinelmer.com/). We performed phylogenetic analysis of *O. tsutsugamushi tsa56* gene sequences and the partial S segment sequences of SFTSV using MEGA7 software (https://www.megasoftware.net/) and constructed phylogenetic trees using the maximum likelihood method (*4*).

References

1. Park SW, Lee CS, Kim JH, Bae IG, Moon C, Kwak YG, et al. Severe fever with thrombocytopenia syndrome: comparison with scrub typhus and clinical diagnostic prediction. BMC Infect Dis. 2019;19:174. PubMed https://doi.org/10.1186/s12879-019-3773-1

2. Ha NY, Kim Y, Min CK, Kim HI, Yen NTH, Choi MS, et al. Longevity of antibody and T-cell responses against outer membrane antigens of *Orientia tsutsugamushi* in scrub typhus patients. Emerg Microbes Infect. 2017;6:1–8. PubMed https://doi.org/10.1038/emi.2017.106

3. Thi Hai Yen N, Kim C, Jeong S, Jeon K, Choi H, Ro HJ, et al. Severe fever with thrombocytopenia syndrome virus infection or mixed infection with scrub typhus in South Korea in 2000–2003. Am J Trop Med Hyg. 2019;101:1096–9. PubMed https://doi.org/10.4269/ajtmh.19-0392

4. Kumar S, Stecher G, Tamura K. MEGA7: Molecular Evolutionary Genetics Analysis, version 7.0 for bigger datasets. Mol Biol Evol. 2016;33:1870–4. PubMed https://doi.org/10.1093/molbev/msw054

Appendix Table 1. Baseline characteristics and the results of serological and molecular diagnosis of suspected scrub typhus patients enrolled in this study

ID	Age (y)	Sex	Province	Fever duration (d)	Eschar	Muscle & joint pain	Rash	Scrub typhus IgG (ICT)		Scrub typhus IFA titer		*tsa56* PCR (genogroup)	SFTS RT-PCR
								TSA56	ScaA	IgG	IgM		
1	10	F	Sagaing	5	–	–	–	+	+	640	640	–	–
2	13	M	Sagaing	4	+	–	–	+	+	10,240	160	–	–
3	2	F	Sagaing	5	+	NA	–	–	–	2,560	80	–	–
4	7	M	Sagaing	4	+	NA	–	+	+	640	–	–	–
5	23	M	Sagaing	5	+	–	–	–	+	2,560	40	–	–
6	3	F	Sagaing	3	+	–	–	–	–	160	160	–	–
7	73	F	Sagaing	4	+	–	–	–	–	640	–	–	–
8	5	M	Sagaing	5	+	–	–	–	–	–	40	–	–
9	12	M	Sagaing	5	+	–	–	+	+	2,560	160	–	–
10	39	M	Sagaing	5	+	–	–	+	+	2,560	–	–	–
11	12	M	Sagaing	15	+	–	–	+	+	640	320	–	–
12	52	M	Sagaing	7	+	NA	NA	–	–	640	–	–	–
13	55	F	Sagaing	5	+	–	–	–	+	10,240	–	–	–
14	10	F	Sagaing	4	+	–	–	+	+	2,560	–	–	–
15	71	F	Sagaing	10	+	NA	NA	–	–	10,240	–	–	–
16	57	F	Sagaing	4	+	–	–	–	–	2,560	640	–	–
17	3	M	Sagaing	7	+	NA	NA	–	–	2,560	–	–	–
18	55	F	Sagaing	2	+	–	NA	+	+	10,240	640	–	–
19	24	F	Sagaing	10	+	+	NA	–	–	640	40	–	–
20	42	F	Sagaing	15	+	+	NA	+	–	640	640	–	–
21	42	M	Sagaing	5	+	–	–	+	+	2,560	160	–	–
22	12	F	Sagaing	4	+	–	–	+	–	640	640	–	–
23	5	F	Sagaing	3	+	+	–	–	–	2,560	160	–	–
24	51	F	Sagaing	6	+	–	–	+	+	10,240	–	–	–
25	15	M	Sagaing	10	+	–	–	–	–	2,560	160	–	–
26	20	M	Sagaing	8	+	–	–	+	+	2,560	–	–	–
27	8	M	Sagaing	3	+	–	–	–	–	2,560	2,560	–	–
28	5	M	Sagaing	3	+	–	–	–	–	160	160	–	–
29	53	M	Sagaing	4	+	–	–	–	–	640	640	–	–
30	6	M	Sagaing	7	–	–	–	–	–	–	160	–	–
31	8	M	Sagaing	3	+	–	–	–	–	40	160	–	–
32	26	F	Sagaing	5	+	–	–	–	–	160	–	–	–
33	55	M	Sagaing	5	+	–	–	–	–	–	–	–	–
34	25	M	Sagaing	7	+	–	+	–	–	160	–	–	–
35	23	F	Sagaing	7	–	+	+	+	–	160	–	–	–
36	23	F	Sagaing	5	+	–	–	+	+	10,240	10,240	–	–
37	19	F	Sagaing	8	–	–	–	–	–	160	–	–	–
38	10	M	Sagaing	3	+	–	–	–	–	–	–	–	–
39	15	M	Sagaing	3	+	–	–	–	–	–	–	–	–
40	7	M	Sagaing	7	+	–	–	–	–	–	160	–	–
41	15	M	Sagaing	7	+	+	–	–	–	–	640	+ (Karp)	–
42	37	M	Sagaing	10	–	–	–	+	+	10,240	–	–	–
43	5	M	Sagaing	3	+	–	–	–	–	–	–	–	–
44	50	M	Sagaing	6	+	+	–	–	–	160	–	–	–
45	11	M	Sagaing	10	+	–	–	–	–	5,120	640	+ (Karp)	–
46	53	F	Sagaing	5	+	–	–	–	+	10,240	–	–	–
47	13	M	Sagaing	5	+	–	–	–	–	10,240	40	–	–
48	68	F	Sagaing	3	+	–	–	–	–	10,240	–	–	–
49	72	F	Sagaing	15	+	–	–	–	–	10,240	–	–	–
50	10	M	Sagaing	4	+	–	–	–	–	10,240	40	–	–
51	16	F	Sagaing	5	+	–	–	–	–	5,120	160	–	–
52	12	M	Sagaing	7	+	–	–	+	+	10,240	2,560	–	–
53	59	M	Sagaing	8	+	–	–	–	–	10,240	–	–	–
54	25	M	Sagaing	8	+	–	–	–	–	1280	–	–	–
55	6	M	Sagaing	2	+	–	–	–	–	640	–	–	–

ID	Age (y)	Sex	Province	Fever duration (d)	Eschar	Muscle & joint pain	Rash	Scrub typhus IgG (ICT)		Scrub typhus IFA titer		*tsa56* PCR (genogroup)	SFTS RT-PCR
								TSA56	ScaA	IgG	IgM		
56	67	M	Sagaing	20	+	–	–	–	–	10,240	640	–	–
57	13	F	Sagaing	3	+	–	–	–	–	320	–	–	–
58	11	F	Sagaing	3	+	–	–	–	–	2,560	–	–	–
59	20	M	Sagaing	5	+	–	–	–	–	10,240	–	–	–
60	13	M	Sagaing	5	+	–	–	–	–	10,240	640	–	–
61	10	F	Sagaing	8	+	–	–	–	–	10,240	10,240	–	–
62	20	M	Sagaing	5	+	–	–	–	–	10,240	–	–	–
63	38	F	Sagaing	4	+	–	+	–	–	5125	–	–	–
64	3	M	Sagaing	2	+	–	–	–	–	80	–	–	–
65	76	M	Sagaing	10	+	–	–	–	–	320	–	–	–
66	26	F	Sagaing	3	+	+	–	–	–	640	160	–	–
67	10	M	Sagaing	5	+	–	–	–	–	320	160	+ (Kato)	–
68	16	F	Sagaing	5	+	–	–	–	–	640	160	+ (Karp)	–
69	30	M	Sagaing	4	+	–	–	–	–	160	–	–	–
70	12	M	Sagaing	4	+	–	–	–	+	2,560	–	–	–
71	17	M	Sagaing	3	+	–	–	–	–	–	–	–	–
72	35	M	Sagaing	3	+	–	–	–	–	10,240	–	–	–
73	14	M	Sagaing	5	+	–	–	–	–	640	–	–	–
74	10	M	Sagaing	5	+	–	–	–	+	10,240	–	–	+
75	4	M	Sagaing	5	+	–	–	NT		160	40	–	+
76	6	M	Sagaing	5	+	–	–	NT		2,560	2,560	–	+
77	42	M	Sagaing	7	–	–	–	NT		10,240	–	–	+
78	7	M	Sagaing	7	+	–	–	NT		10,240	640	–	–
79	59	F	Sagaing	5	+	+	–	NT		10,240	–	–	–
80	40	M	Sagaing	5	+	–	–	NT		–	160	–	–
81	14	M	Sagaing	5	+	–	–	NT		–	40	–	+
82	5	M	Sagaing	5	+	–	–	NT		2,560	10,240	–	–
83	60	F	Sagaing	5	–	–	–	NT		2,560	–	–	–
84	16	M	Sagaing	5	+	–	–	NT		640	2,560	–	–
85	9	M	Sagaing	5	+	–	–	NT		2,560	2,560	–	–
86	20	F	Sagaing	10	+	–	–	NT		2,560	10,240	–	–
87	10	M	Sagaing	3	+	–	–	NT		2,560	2,560	+ (Gilliam)	–
88	50	F	Sagaing	7	+	–	–	NT		5,120	–	–	–
89	41	M	Sagaing	5	+	–	–	NT		160	–	–	–
90	13	M	Sagaing	3	+	–	–	NT		–	–	–	–
91	11	M	Sagaing	5	+	–	–	NT		40	–	–	–
92	10	M	Sagaing	4	+	–	–	NT		160	–	–	–
93	29	M	Sagaing	5	+	–	–	NT		–	–	+ (Karp)	–
94	10	M	Sagaing	5	+	–	–	NT		160	–	–	–
95	5	F	Sagaing	NA	+	–	–	NT		–	–	–	–
96	63	F	Sagaing	5	+	–	–	NT		–	–	–	–
97	5	F	Sagaing	5	+	–	–	NT		640	2,560	–	–
98	9	M	Sagaing	5	+	–	–	NT		640	10,240	–	–
99	10	M	Sagaing	5	+	–	–	–	–	–	40	+ (Kato)	–
100	12	M	Sagaing	4	+	–	–	+	+	10,240	10,240	–	–
101	12	F	Sagaing	10	+	–	–	+	–	2,560	2,560	–	–
102	50	M	Sagaing	5	+	–	–	+	–	640	–	–	–
103	45	F	Sagaing	5	+	–	–	–	–	40	–	–	–
104	40	F	Sagaing	6	+	NA	–	–	–	40	40	–	–
105	26	M	Sagaing	3	+	–	–	–	–	160	–	–	–
106	42	M	Sagaing	10	+	–	–	+	–	2,560	–	–	–
107	12	F	Sagaing	5	+	–	–	–	–	160	160	–	–
108	19	F	Sagaing	3	+	–	–	+	–	640	640	–	–
109	10	F	Sagaing	4	+	–	–	–	–	–	80	–	–
110	36	M	Sagaing	2	+	–	–	–	–	–	40	–	–
111	15	M	Sagaing	7	+	–	–	–	–	–	–	–	–
112	19	M	Sagaing	7	+	+	–	–	–	–	2,560	–	–
113	12	M	Sagaing	7	+	–	–	–	–	–	10,240	–	–
114	21	M	Sagaing	5	+	–	–	+	–	640	160	–	–
115	11	F	Sagaing	3	+	–	–	–	–	40	2,560	–	–
116	29	M	Sagaing	5	+	+	–	–	–	40	640	–	–
117	33	M	Sagaing	5	+	–	–	–	–	–	80	–	–
118	11	M	Sagaing	7	–	–	–	+	+	640	2,560	–	–
119	6	F	Sagaing	3	+	–	–	+	+	160	160	–	–
120	21	M	Sagaing	5	+	–	–	–	–	–	–	–	–
121	8	F	Sagaing	5	+	–	–	+	–	80	10,240	–	–
122	54	M	Sagaing	7	+	–	–	+	+	160	640	–	–

ID	Age (y)	Sex	Province	Fever duration (d)	Eschar	Muscle & joint pain	Rash	Scrub typhus IgG (ICT)		Scrub typhus IFA titer		*tsa56* PCR (genogroup)	SFTS RT-PCR
								TSA56	ScaA	IgG	IgM		
123	66	M	Sagaing	10	+	–	–	+	+	640	–	–	–
124	62	F	Sagaing	12	+	–	–	+	+	640	1280	–	–
125	43	F	Sagaing	7	+	–	–	–	–	–	–	–	–
126	29	M	Sagaing	4	+	–	–	+	–	80	–	–	–
127	8	M	Sagaing	3	+	–	–	–	–	40	160	–	–
128	14	M	Sagaing	5	–	–	–	–	–	40	640	–	–
129	21	F	Sagaing	5	+	–	–	–	–	640	2,560	+ (Gilliam)	–
130	25	F	Magway	7	+	–	–	–	–	–	640	–	–
131	11	F	Magway	5	+	–	–	–	–	160	10,240	–	–
132	33	F	Magway	8	+	+	–	–	–	–	40	–	–
133	44	F	Magway	7	+	–	–	–	–	–	–	–	–
134	52	F	Magway	9	+	–	–	–	–	–	40	–	–
135	50	F	Magway	10	+	+	–	–	–	640	10,240	–	–
136	50	F	Magway	9	+	+	–	–	–	2,560	640	–	–
137	45	M	Magway	5	+	+	–	+	–	10,240	2,560	–	–
138	62	M	Magway	10	+	–	–	+	–	10,240	10,240	–	–
139	24	M	Magway	7	+	+	–	–	–	–	10,240	–	–
140	40	F	Magway	10	+	+	–	–	–	40	80	–	–
141	50	F	Magway	10	+	+	–	+	–	–	2,560	–	–
142	44	F	Magway	10	+	+	–	–	–	640	10,240	–	–
143	30	M	Magway	8	+	–	–	–	–	640	10,240	–	–
144	26	M	Magway	10	+	+	–	–	–	160	640	+ (Karp)	–
145	53	F	Magway	7	+	+	–	–	–	–	640	–	–
146	55	M	Magway	15	+	+	–	+	–	640	10,240	–	–
147	23	M	Magway	6	+	+	–	–	–	80	640	–	–
148	22	M	Magway	6	+	+	–	+	–	–	–	–	–
149	30	M	Magway	10	+	–	–	–	–	–	40	–	–
150	57	M	Magway	12	+	+	–	–	–	40	10,240	–	–
151	46	M	Magway	7	+	+	–	+	–	160	2,560	–	–
152	5	F	Magway	7	+	–	–	–	–	40	10,240	–	–

ICT, immunochromatography test; IFA, immunofluorescence assay; NA, not available; NT, not tested; RT, reverse transcription; SFTS, severe fever with thrombosis syndrome; +, positive result; –, negative result

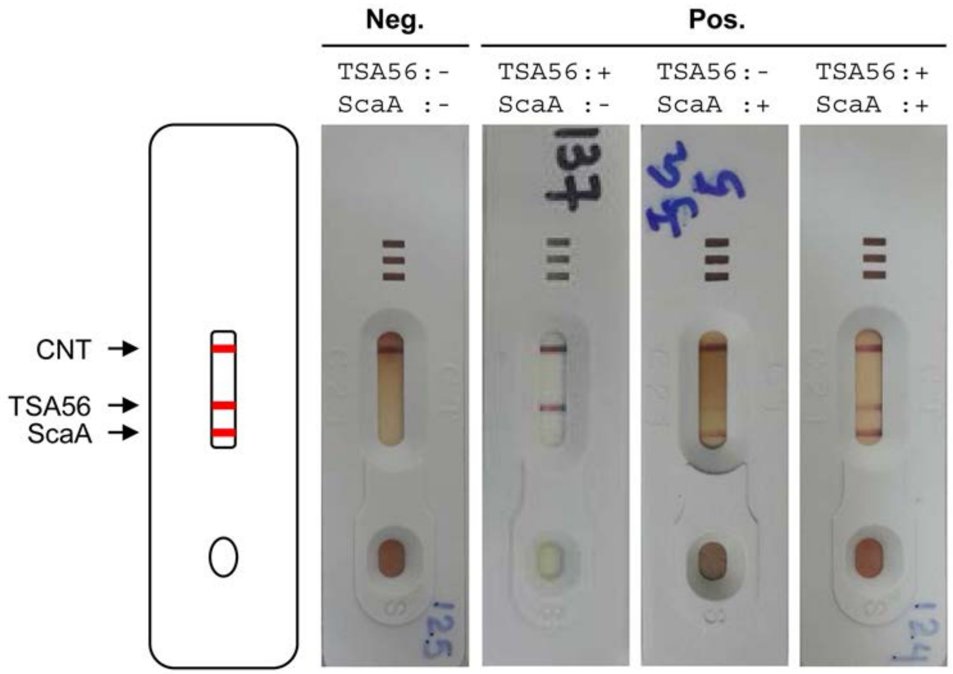

Appendix Figure 1. Representative images of ICT results detecting specific IgG against TSA56 and ScaA antigen in suspected scrub typhus patients' sera.

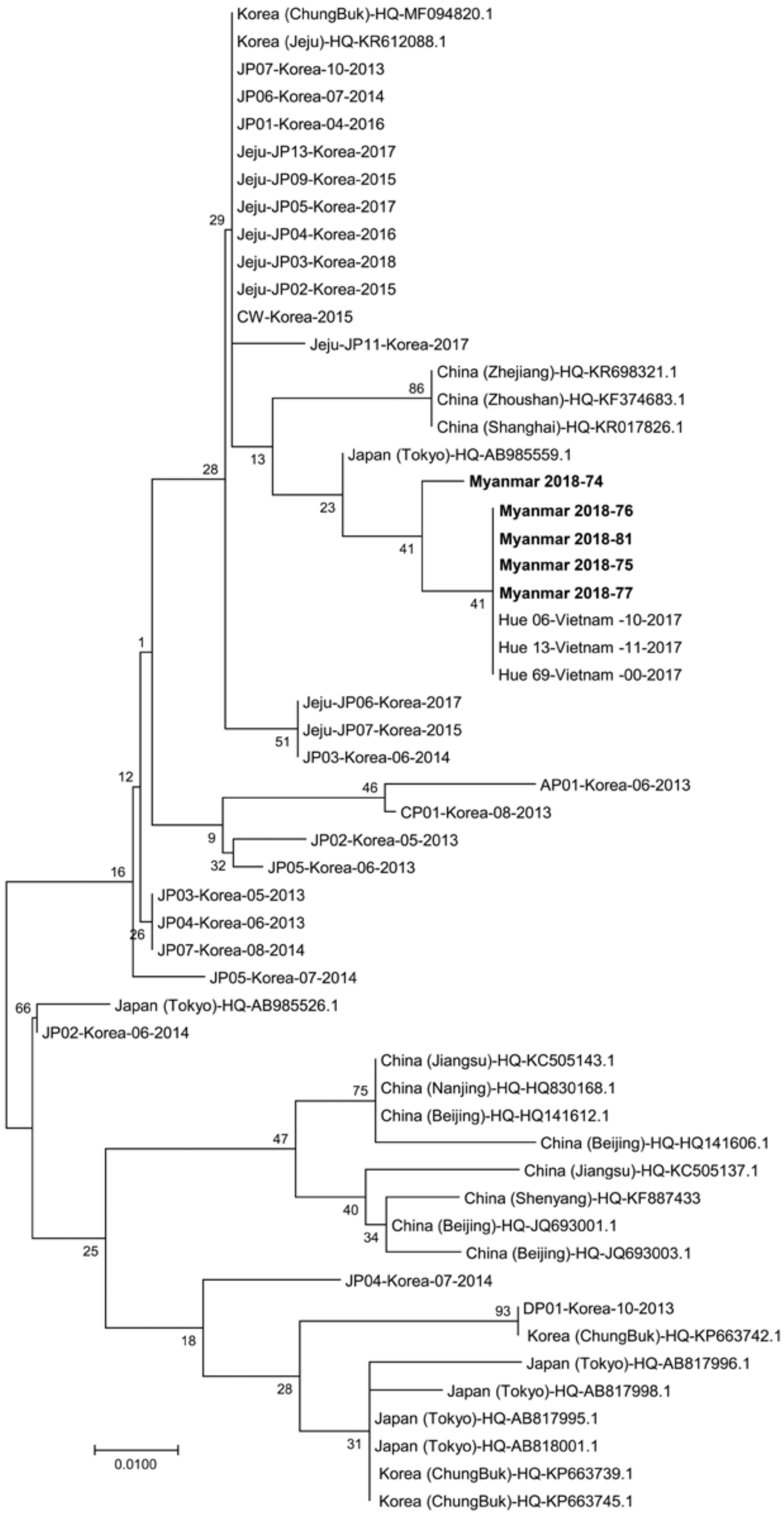

Appendix Figure 2. Phylogenetic tree constructed based on partial S segment sequences of SFTSV. The tree was constructed using the maximum likelihood method with MEGA7. The partial S segment sequences amplified from the serum samples of indicated patients were analyzed and are shown in red.
